# Influence of sexually transmitted infections on the cervical cytological abnormalities among Iranian women: A cross-sectional study

**DOI:** 10.18502/ijrm.v21i6.13636

**Published:** 2023-07-24

**Authors:** Azam Shafaei, Seyyed Ali Akbar Shamsian, Mohammad Ghodsi, Fatemeh Sadabadi, Maryam Shahi

**Affiliations:** ^1^Blood Borne Infections Research Center, Academic Center for Education, Culture and Research (ACECR), Razavi Khorasan Branch, Mashhad, Iran.; ^2^Department of Parasitology and Mycology, Faculty of Medicine, Mashhad University of Medical Sciences, Mashhad, Iran.; ^3^Clinical Laboratory Diagnostic Center, Academic Center for Education, Culture and Research (ACECR), Khorasan Razavi, Mashhad, Iran.; ^4^Cutaneous Leishmaniasis Research Center, School of Medicine, Mashhad University of Medical Sciences, Mashhad, Iran.

**Keywords:** Sexually transmitted infections, Human papillomavirus, Cervical cancer, Genital wart, Genotypes.

## Abstract

**Background:**

Sexually transmitted infections (STIs) are one of the world's most severe health challenges. The existence of STIs such as human papillomavirus (HPV) might cause cervical cell changes leading to cervical cancer.

**Objective:**

This study aims to assess the association of STIs with cervical cytological abnormalities and genital warts among women in northeastern Iran.

**Materials and Methods:**

This cross-sectional study was carried out on 190 women referred to the central laboratory of Academic Center for Education, Culture, and Research, Mashhad, Iran from March to July 2022. The presence of genital infections caused by *Chlamydia trachomatis*, *Neisseria gonorrhoeae*, *Mycoplasma genitalium*, and Herpes simplex viruses (1 and 2) were assessed using the real-time polymerase chain reaction method. HPV genital infection was detected based on the principles of reverse hybridization, and cellular changes in the cervix were examined by the liquid-based cytology technique.

**Results:**

The mean age of participants was 35.33 ± 8.9 yr. 34 different HPV genotypes were detected in all HPV-positive cases, and the most common genotype was low-risk HPV6. No significant association was found between STIs and cervical cytology abnormalities. The prevalence rates of sexually transmitted pathogens among HPV-positive and HPV-negative individuals were 10.9 and 1.6%, respectively. The frequency of genital warts was significantly higher in cases with multiple infections of high- and low-risk HPV genotypes.

**Conclusion:**

High percentages of the participants with non-HPV STIs and HPV infection had normal cervical cytology. It is advised to use STIs and HPV diagnostic tests along with cytology examinations for cervical cancer screening.

## 1. Introduction

Sexually transmitted infections (STIs) are considered a global health problem (1). There are > 30 types of STIs caused by several pathogens, including viral agents (such as Herpes simplex virus type 2 [HSV-2] and human papillomavirus [HPV]), genital bacterial infections (such as *Chlamydia trachomatis *[*C. trachomatis*]*, Neisseria gonorrhoeae *[*N. gonorrhoeae*], and *Mycoplasma spp*.), and protozoa (*trichomonas vaginalis*) (2). More than 1 million people are infected with STIs per day (3).

Most STIs infection are asymptomatic and can lead to various outcomes, such as pelvic inflammatory disease, sterility, ectopic pregnancy, congenital infections, and cervical cancer (4). The presence of some STIs facilitates the transmission of other infections and can cause cellular changes that precede some types of cancer (5). Cervical cancer has been one of the most common cancers among females and the 4 ${}^{\text{th}}$ most common type after breast, colorectal, and lung cancers. It was shown that an important cause of cervical cancer is HPV (6).

HPV is one of the most frequent STIs and is normally transmitted through direct contact with infected skin or mucosa (7). Depending on their oncogenic potential, HPV is divided into high-risk (HR) and low-risk (LR) groups. This should be mentioned that the HPV infection is effective risk factor for cervical cancer but it is not enough cause of cancer (8). Most HPV infections are temporary; however, a few cases of infections continue and progress to HR lesions and cancer.

Some STIs agents such as *N. gonorrhoeae* and *Mycoplasma genitalium* (*M. genitalium*) could change the genital tract flora through repeated infections and provoke cervical cancer. The increase in microbial species in genital tract causes an increase in cervical cancer (9, 10). Determining the presence of STIs is essential for developing appropriate healthcare strategies that focus on preventive and protective measures to reduce the risk of transmission and their outcomes. The current status of co-infections among Iranian communities is not well known.

Therefore, the purpose of the present study was to assess the association between STIs (*C. trachomatis*, *N. gonorrhoeae*, *M. genitalium*, HSV-1/2, and HPV) with cervical cytological abnormalities and genital warts among Iranian women referred to the central laboratory of Academic Center for Education, Culture, and Research, Mashhad Branch, Iran.

## 2. Materials and Methods 

### Study population and design

This cross-sectional study was carried out on 190 women aged between 16 and 68 yr who were referred to the central laboratory of Academic Center for Education, Culture, and Research, Mashhad, Iran between March and July 2022. Demographic characteristics and lifestyle (age, smoking, and history of abortion) were obtained from the available medical records. Any of the following factors were considered as exclusion criteria: pregnancy, being in the postpartum period, and having no history of sexual activity.

### Evaluation of cervical cytology 

Cervical cytology samples were collected for the liquid-based cytology technique to assay cellular changes. The cervical cytology of each participants was evaluated. The stages of the cervical cytology are classified as negative for squamous intraepithelial lesion or malignancy (NILM), atypical squamous cells (ASC) of undetermined significance (US), ASC-high-grade, low-grade squamous intraepithelial lesions (LSIL), high-grade squamous intraepithelial lesions, and invasive cervical cancer.

### DNA extraction and molecular diagnosis

The cervical samples were used in molecular experiments for HPV and STIs detection. DNA was extracted from cervical cytology samples using SSNP-2000B nucleic acid extraction system produced by Bioperfectus, China. The DNA solution was stored at -20 C for HPV and STI tests.

The HPV DNA test was performed using a high + low papillomastrip kit (OPERON, Spain) according to the manufacturer's protocol. This kit allows the identification of 37 HPV subtypes, including HR: 16, 18, 26, 31, 33, 35, 39, 45, 51, 52, 53, 56, 58, 59, 66, 68, 69, 73, and 82, and LR: 6, 11, 40, 42, 43, 44, 54, 61, 62, 67, 70, 72, 74, 81, 83, 84, and 91. For STIs detection, real-time PCR with REALQUALITY RQ-STI CT/NG/MG Kit (AB-Analitica, Italy) was used according to the manufacturer's instructions.

The kit includes an internal control consisting of a recombinant DNA fragment of the β -*globin* gene. It allows verification of the extraction procedure and detection of PCR inhibition. Real-time PCR was applied for HSV determination using the HSV1/2 RQ kit (Novingene, Iran).

### Ethical considerations

Written informed consent was obtained from all participants according to the protocol approved by the Ethics Committee of Academic Center for Education, Culture, and Research, Razavi Khorasan Branch, Mashhad, Iran (Code: IR. ACECR.JDM.REC.1401.065).

### Statistical analysis

Data analysis was performed using Statistical Package for the Social Sciences, version 22, SPSS Inc., Chicago, Illinois, USA (SPSS). Descriptive analyses including frequencies, percentages, and averages were used to describe the population under study. A Chi-square test or Fisher's exact test and regression analysis were performed for the qualitative variables when deemed necessary. Binary logistic regression was used to evaluate the independent effects of smoking on HPV infection. The statistical significance was set at p < 0.05.

## 3. Results

A total of 190 women participated in this study, including 128 HPV-positive and 62 HPV-negative women as the control group. The demographic characteristics of the participants are shown in table I. The mean age of the participants was 35.33 ± 8.9 yr (16-68 yr).

The mean age of the HPV-positive women was 34.60 ± 8.8 yr (16-56 yr) and the mean age of the HPV-negative-women was 36.82 ± 9.2 (22-68 yr), and no significant difference was observed between the 2 groups. 34 different HPV genotypes were detected in all the HPV-positive women. The 7 most common genotypes were LR-HPV6 (26.6%), HR-HPV16 (19.5%), HR-HPV52 (12.5%), LR-HPV54 (10.9%), LR-HPV91 (10.2%), LR-HPV11 (9.4%), and LR-HPV42 (8.6%). HR-HPV prevalence was 83/128 (64.84%).

LR-HPV6 was the most common genotype in women with mono-infection with n = 12/68 (17.6%) frequency followed by HR-HPV16 with n = 9/68 (13.2%) and LR-HPV42 with n = 4/68 (5.9%). Furthermore, in women with multiple infections, LR-HPV6 with a frequency of n = 22/60 (36.7%) was more prevalent than other genotypes followed by HR-HPV16 n = 16/60 (26.7%) and HR-HPV52 n = 15/60 (25%). Many of the HPV-positive women were infected with multiple HPV genotypes. In other words, we detected n = 60/128 (46.87%) multiple HPV genotypes in the total participants.

No significant association was observed between HPV infection and age in classified age groups, age of marriage in classified marriage age groups, history of abortion, and level of education, as shown in table I. Furthermore, no significant correlation was observed between HPV infection and abnormal cervical cytology, (Table I). Our results showed that smoking is involved in increasing the prevalence of HPV infection. The prevalence of STIs pathogens was significantly different betweenHPV-positive and HPV-negative participants. The prevalence rate of STIs pathogens in the HPV-positive participants was 10.9% (14/128) and it was 1.6% (1/62) in the HPV-negative participants (Table I). *C. trachomatis*, *M. genitalium*, and HSV1/2were the STIs detected in this study.

The most common pathogen was *M. genitalium* (n = 6, 4.6%) followed by *C. trachomatis* (n = 4, 3.1%) and HSV1/2 (n = 4, 3.1%) in the HPV-positive individuals. The most observed Pap smear results were NILM 176/190 (92/6%). Abnormal cervical cytology was detected in 14/190 (7.3%) of the participants, including 10 women with ASC-US (5.2%) and 4 with LSIL (2.1%). All 4 LSIL participants were HPV positive and 7 of the ASC-US individuals were HPV positive. HPV was detected in n = 117/176 (66.5%) women with NILM cytology. Among women with abnormal cytology, n = 11/14 (78.6%) were HPV positive (Table II). However, no significant association was observed between HPV infection and abnormality of cervical cytology. Moreover, no association was found between HPV genotypes (HR and LR) and abnormality of cervical cytology. In addition, STIs or co-infection of HPV and STIs had no significant relationship with an abnormality of cervical cytology (Table II).

Based on table III, type of HPV (LR, HR or high and low risk) infection is associated with the incidence of warts. The frequency of warts is higher in people who have multiple infections of HR and LR genotypes, and it is more common in people who have an LR-HPV genotype compared to cases with HR-HPV. In addition, abnormal cervical cytology in people with multiple infections of HR and LR genotypes was higher than in the mono-infection group, but this difference was not significant (p = 0.2).

LR-HPV6 was the most common genotype in wart sufferers, and it was detected in 25/68 (36.76%) of these women, 11 of whom were identified as having mono-infection and 12 had multi-infection with one or more HR-HPV genotypes while 2 participants showed multi-infection with another LR-HPV genotype. The second prevalent genotype in wart sufferers was HR-HPV16 with a frequency of 10/68 (14.70%), 6 of whom had multi-infection with one or more LR-HPV genotypes.

In this study, it was found that the risk of HPV infection increased 5.43 times in the current-smoker cases compared to non-smokers (OR = 5.43, CI 95% (1.56-18.85), p < 0.01).

**Table 1:** Demographic and clinical characteristics of HPV negative and positive subjects


**Variables **		**HPV-negative (n = 62)**	**HPV-positive (n = 128)**	**P-value**
**Age (yr)**				
	** ≤ 25**	4 (21.1)	15 (78.9)	
	**26-35**	26 (32.5)	54 (67.5)	
	** > 35**	32 (35.2)	59 (64.8)	0.49
**Marriage age (yr)**				
	** ≤ 18**	19 (30.6)	55 (43)		
	**19-28**	37 (59.7)	57 (44.5)	
	**> 28**	6 (9.7)	16 (12.5)	0.15
**Smoking status**				
	**Non-smoker**	59 (36.9)	101 (63.1)		
	**Current smoker**	3 (10)	27 (90)	< 0.001
**History of abortion**				
	**No abortion**	42 (67.7)	93 (72.7)	
	**Single abortion**	14 (22.6)	22 (17.2)	
	**Multiple abortions**	6 (9.7)	13 (10.2)	0.67
**Educational level**				
	**Secondary school**	16 (25.8)	25 (19.5)		
	**High school **	22 (35.5)	44 (34.4)	
	**Academic education**	24 (38.7)	59 (46.1)	0.53
**Genital wart**				
	**Negative**	59 (95.2)	63 (49.2)		
	**Positive**	3 (4.8)	65 (50.8)	< 0.001
**Non-HPV STIs**				
	**Negative**	61 (98.4)	114 (89.1)	
	**Positive**	1 (1.6)	14 (10.9)	0.02
**Cervical cytology findings**				
	**Normal (NILM)**	59 (95.2)	117 (91.4)	
	**Abnormal**	3 (4.8)	11 (8.6)	0.40
Data presented as n (%). Chi-square test. HPV: Human papillomavirus, STIs: Sexually transmitted infections, NILM: Negative for squamous intraepithelial lesion or malignancy				

**Table 2:** Prevalence of HPV, HR-HPV, LR-HPV, STIs, and HPV/STIs by different cervical cytology findings


	**Cervical cytology findings**			
**Variable**	**Normal (NILM) (n = 176)**	**ASC-US (n = 10)**	**LSIL (n = 4)**	**P-value**
	**HPV-positive**	117 (66.5)	7 (70)	4 (100)	0.51
**HR-HPV**	40 (22.7)	0 (0)	2 (50)	
**LR-HPV**	42 (23.9)	3 (30)	0 (0)	
**HR & LR HPV**	35 (19.9)	4 (40)	2 (50)	0.08
**STIs-positive**	14 (8)	1 (10)	0 (0)		0.70
**HPV/STIs**	13 (7.3)	1 (10)	0		0.67
Data presented as n (%). Chi-square test. HPV: Human papillomavirus, NILM: Negative for squamous intraepithelial lesion or malignancy, ASC-US: Atypical squamous cells of undetermined significance, LSIL: Low-grade squamous intraepithelial lesions, HR-HPV: High-risk HPV, LR-HPV: Low-risk HPV, HR/LR HPV: Co-infection of HR and LR HPV, STI: Sexually transmitted infections, HPV/STIs: Co-infection of HPV and STIs				

**Table 3:** Prevalence of different HPV infection types among individuals with the genital wart


	**HPV positive**			
**Wart**	**HPV negative**	**LR-HPV**	**HR-HPV**	**HR- & LR-HPV**	**Total**	**P-value**
		**Negative**	59 (48.4)	18 (14.7)			32 (26.2)	13 (10.7)	122 (64.21)	
**Positive**	3 (4.4)	27 (39.7)	10 (14.7)	28 (41.2)	68 (35.78)	
**Total**	62 (32.6)	45 (23.7)	42 (22.1)	41 (21.6)	190	< 0.001
Data presented as n (%). Chi-square test. HPV: Human papillomavirus, LR-HPV: Low-risk HPV, HR-HPV: High-risk HPV						

## 4. Discussion

In the present study, 34 different HPV genotypes were detected in all HPV-positive women. The most common genotype was LR-HPV6 (26.6%). We detected 3 important STIs including *C. trachomatis*, *M. genitalium*, and HSV1/2 in 10.9% of HPV-positive participants; however, no significant association was found between the cervical cytological abnormalities and STIs positive in HPV positive group. Because of the low frequency of co-infections between HPV and other STIs, we were unable to make a proper evaluation of the influence of this association in abnormal cytology results. In our study, it was found that the most positive women for HPV and STIs were women ≤ 25 yr of age. Similar to our result, another study has shown a higher HPV prevalence in 25 yr women or less, which later decreases obviously in middle-aged women. This finding is consistent with another study that has recommended that HPV and non-HPV STIs are more prevalent in young women ≤ 25 yr of age (11). Perhaps immature cervical tissue and higher sexual activity make these women prone to the growth of pathogens (12). One of the cofactors associated with HPV infection is the age of the first sexual intercourse (11), but no significant difference was detected between marriage age and HPV or non-HPV STIs in the current study. Due to cultural and traditional restrictions, it is not convenient for many people to reveal the time of their first intercourse, so this question was replaced with marriage age, which includes errors. The most prevalent genotypes identified in the current study were LR-HPV6 (26.6%) followed by HR-HPV16 (19.5%). Notably, LR-HPV6 was prevalent in both mono-infection HPV and multi-infection HPV. An investigation was consistent with our results and showed the frequency of LR-HPV6 and HR-HPV16 to be 77 and 15%, respectively, as the most prevalent genotypes, while HPV-18 was not detected in their study (13).

In contrast to the present results, a study found that HR-HPV16 was the most prevalent genotype (49.2%) followed by HR-HPV18 (25.3%), while in the current work, the prevalence of HR-HPV18 was very low (2.3%) (2). In a previous study conducted by the authors, the LR-HPV6 was the most prevalent genotype (43.9%) and HR-HPV18 was not prevalent in comparison with other genotypes with a frequency of 17.7%, which is considerably higher than its prevalence in the current study. This may be due to changes in the trend of prevalent genotypes in the region under study (14). The frequency of multiple HPV-genotype infections was relatively high in the current study (46.87%). One study showed that multiple HPV infections were associated with the occurrence of cytological abnormality (11), which is consistent with this study. Moreover, a significantly higher prevalence of warts was found in participants with multiple HPV infections, and more cytological abnormalities were identified in participants with multiple HPV infections in this study, but they were not significant.

*C. trachomatis* may be a reason for female infertility and clinical manifestations (15). *C. trachomatis* is a prevalent sexually transmitted bacterial infection (16). Nevertheless, the most common pathogen in our study was *M. genitalium* followed by *C. trachomatis* and HSV. In the meta-analysis comprised of 34 studies in Iran in a period from 1998-2015, it was demonstrated that the prevalence of *C. trachomatis* in women and men was 0-32.7 and 0-23.3%, respectively. The differences were related to different geographical regions, methods of detecting microorganisms, and sample size (15). An investigation that had results similar to our study, detected only one infection with *C. trachomatis *(0.84%) in the HPV-positive group (17). The rate of HPV infections in cases with abnormal cervical cytology was high (78/6%) in the current study. Another study which had similar findings, detected 78.8% of HPV infections in cervical cancer samples (18).

In contrast to the results of another study, no significant association was found between HPV infection or STIs and cervical cytological abnormalities in the present study (2). In a study with similar results to the current study, no significant association was observed between HPV/STIs co-infection and the cervical abnormality (19). In agreement with other studies, our results demonstrated an association between HPV infection and the incidence of warts.

We found LR-HPV6 to be the most common genotype among wart sufferers. In an investigation that had similar results to this work, they showed that LR-HPV6 was the most prevalent genotype in wart samples (36.3%) (20). In this research, it was revealed that multiple infections of HR and LR genotypes are more likely to cause warts, and warts are more common in people who have an LR-HPV genotype than in cases with HR-HPV. Consistent with the current results, another study revealed that co-infection of both low- and HR-HPV types increases the risk of developing genital warts (OR: 2.814; 95%: 1.208-6.55, p = 0.017). Additionally, similar to the results of the present study, they found that LR-HPV genotypes have a higher risk of genital warts in comparison with HR-HPV types (21). The results of a study showed that the LR-HPV genotype was the major cause of genital warts and HR-HPV infections, and multiple HPV-type co-infections were also common in genital warts. Also, they found HPV6, 11, 52, and 16 as the 4 most common HPV types in genital warts (22). In the current study, HR-HPV16 was the 2 ${}^{\text{nd}}$ most prevalent genotype in participants with warts.

Similar to the present results, a study demonstrated that the most prevalent genotype was LR-HPV6 (47%), but unlike the current study, in which HR-HPV16 was detected as the 2 ${}^{\text{nd}}$ most prevalent cause of wart, they identified LR-HPV11 (13.6%) as the 2 ${}^{\text{nd}}$ most widespread genotype (23).

As revealed in another study as well as the present study, the risk of HPV infection increases in current smoker individuals in comparison with non-smokers. Similar to the findings of this research, they showed that the risk of HPV infection increases 1.9 times from smokers to non-smokers (OR = 1.905, CI 95% (1.426-2.545), p < 0.05) (24).

## 5. Conclusion

In conclusion, the current study has shown a high prevalence of LR-HPV6 in HPV-positive individuals as well as in cases with warts. It was found that smoking increases the risk of HPV infection. A high percentage of HPV-positive and STI-positive participants had normal Pap smear, so it seems that a Pap smear test alone is not enough for cervical cancer screening, and it is suggested to prepare HPV and non-HPV STI tests as well. According to the present results, higher prevalence rates of HPV and STI were found in women ≤ 25 yr of age, and it seems that a STI screening plan should be applied at this age.

The potential limitations of the current study were sampling method and small sample size. Since the number of participants with abnormal cytology was very small in the current study, more investigations are needed with larger sample sizes in a specific population of women with abnormal cytology.

##  Conflicts of Interest 

The authors declare that they have no conflict of interest.
